# Development and pilot evaluation of a complex intervention to improve experienced continuity of care in patients with cancer

**DOI:** 10.1038/sj.bjc.6604836

**Published:** 2008-12-23

**Authors:** M King, L Jones, O McCarthy, M Rogers, A Richardson, R Williams, A Tookman, I Nazareth

**Affiliations:** 1 Department of Mental Health Sciences, Royal Free and University College Medical School, Royal Free Campus, Rowland Hill Street, London NW3 2PF, UK; 2 Marie Curie Palliative Care Research Unit, Department of Mental Health Sciences, Royal Free and University College Medical School, Royal Free Campus, Rowland Hill Street, London NW3 2 PF, UK; 3 King's College London, Florence Nightingale School of Nursing and Midwifery, Waterloo Bridge Wing, Franklin Wilkins Building, Stamford Street, London SE1 8WA, UK; 4 MRC General Practice Research Framework and Department of Primary Care and Population Science, Royal Free and University College Medical School, Royal Free Campus, Rowland Hill Street, London NW3 2PF, UK

**Keywords:** continuity of care, complex intervention, feasibility trial

## Abstract

High experienced continuity of care in patients with cancer is associated with lower needs for care, better quality of life and better psychological outcomes. We developed and evaluated an intervention to improve experienced continuity. The intervention, consisted of (1) a 17-item patient-completed continuity assessment; (2) feedback to clinical nurse specialists and action to address the needs identified. Multidisciplinary team meetings and oncology outpatient clinics were observed, and patients and staff were interviewed. After qualitative work and reliability testing, the intervention was evaluated in a feasibility trial. Sixty-one patients provided data for analysis. No statistically significant differences were found in patients' experienced continuity between the trial arms, but important trends were seen in measures of needs for care in favour of those receiving the intervention. Feeding back findings from the continuity assessment to clinicians reduced patients' needs for care. Our results indicate that an intervention to target patients' experiences of continuity can reduce their subsequent needs for care. However, overcoming barriers to organisational change and addressing some patients' hesitation to report their continuity difficulties must be considered when implementing such an intervention. A phase III trial targeting patients with inadequate experienced continuity of care is recommended.

As survival rates for cancer improve, continuity of care in patients with cancer is becoming a research priority (Department of Health, 2007). The Department of Health in England has recently launched the Cancer Survivorship Initiative, and continuity of care is one of the key themes to be explored (Department of Health, 2008). Many cancers are now experienced as a chronic disease and survivors may fear recurrence, feel out of touch with service providers and experience feelings of uncertainty. The National Institute for Clinical Excellence's guidance on Supportive and Palliative Care (NICE, 2004) recommended promoting mechanisms to enhance continuity of care, and observed that few studies have researched the ‘impact of continuity of care on the process and outcomes of care’.

In earlier research, we used a combination of methods to identify the importance of experienced continuity for patients with breast, lung and colorectal cancer at all phases of disease (King *et al*, 2008). We concluded that continuity is a concept best defined by how it is experienced by patients rather than how it is delivered by services. We also found that higher experienced continuity is associated with lower future needs for supportive care and better psychological outcomes. This research was part of a wider programme commissioned by NIHR Service and Delivery Organisation to explore concepts of continuity of care in chronic disease. Findings from the programme were synthesised and combined with evidence from the international literature, in particular from work conducted in Canada (Freeman *et al*, 2007). The analysis concluded that patients should be encouraged to behave, as partners in their care and professionals should aim to work with patients rather than simply delivering a service to them. Few studies have attempted to evaluate mechanisms for improving continuity of care in cancer. These have focussed largely on service delivery and attempted to improve co-ordination of care through new care models to join up services (Smeenk *et al*, 1998), provision of additional support from secondary to primary care professionals (Johansson *et al*, 1999) and the introduction of nurse co-ordinators for patients in the last year of life (Addington-Hall *et al*, 1992). None of these interventions has shown patient benefit.

Continuity as experienced by patients may incorporate the three elements of continuity (informational, management and relationship) described previously (Haggerty *et al*, 2003). Recent development of care pathways and guidelines may improve informational and management continuity, but will not influence relationship continuity (Guthrie *et al*, 2008).

We used our findings to formulate an intervention to improve continuity of care that was patient centred. In this study, we report on further development of this intervention and its performance in a feasibility randomised controlled trial. The intervention consisted of two parts: (1) a patient completed 17-item continuity assessment and (2) feedback to staff of patients' responses to the assessment.

## Materials and methods

### Development of the intervention

This study was conducted in breast, lung and colorectal cancer services at four North London NHS Trusts. Additionally, two palliative clinics and two multidisciplinary team meetings were observed at a North London Marie Curie Hospice. Ethical approval was obtained from East London and the City Multisite Research Ethics Committee 1 on 23 May 2006.

We observed clinics and multidisciplinary team meetings and conducted semistructured interviews with staff and patients to:

1. Inform the timing of, the people with whom, and the context within which the intervention would be delivered.
2. Obtain professionals' and patients' views on the acceptability and practicability of delivering and receiving the intervention.

#### Observations of clinics and multidisciplinary team meetings

Our initial plan was for each patient's experienced continuity to be reviewed at multidisciplinary team meetings. Two project researchers explored the feasibility of this idea by observing breast, lung and colorectal multidisciplinary team meetings and outpatient clinics. Our objectives were to observe the content of the multidisciplinary team meetings and interactions between team members. We focused on patient-centred issues (e.g., their history or social circumstances), which we knew were likely to impact on continuity of care (King *et al*, 2008). We considered the possible content and timing of a continuity assessment and how it might be presented at the multidisciplinary team meeting. Our observations of clinic consultations and staff meetings aimed to identify how gaps in continuity of care were identified and managed by staff.

#### Staff interviews

We conducted semistructured interviews with five clinical nurse specialists (CNS), three consultant medical oncologists, two specialist registrars and two research nurses all of whom worked in services for the three cancer types at the participating NHS Trusts. The interviews focused on the structure, content and format of the proposed intervention, the possible timing and setting of its delivery, and which member of staff might be best placed to undertake it. We also asked participants to consider how they might manage issues arising from the continuity assessment.

#### Patient interviews

We assessed the views of patients with breast, lung or colorectal cancer on the structure and content of the continuity assessment. We approached outpatients opportunistically in each of the five phases of cancer treatment: recently diagnosed, following initial treatment, remission, recurrence and palliative care. After giving informed consent, patients completed the continuity assessment without assistance and were then interviewed in private to discuss the structure and content of the questionnaire. To assess test–retest reliability of the assessment, patients were asked to complete the questionnaire again at home after 2 weeks and return it by post.

#### Iterations of the continuity assessment

The continuity assessment was developed through two iterations. Version 1 consisted of a continuity assessment composed of four domains that we had developed in earlier work (King *et al*, 2008). We had planned that the assessment would be completed by clinicians and presented at the multidisciplinary team meeting to inform team decision making. However, because nurses felt strongly that they had insufficient time to help patients complete the assessments, we concluded that the continuity assessment should be entirely patient focussed. Thus, we developed a second version for patient self-completion that contained 18 items from our earlier work (King *et al*, 2008), and six additional items from Version 1. Responses to patient's first and second assessments were entered into STATA release 9 and analysed using the *κ*-statistic. Items with low reliability were dropped, which resulted in 17 items in a final version.

#### Continuity intervention tested in the feasibility trial

The intervention had two components:

1. Component 1: The patient-completed 17 item continuity assessment (Box 1). After each item, patients ticked a box if they wished to discuss the issue further with a clinical team member. Four boxes were given at the end of the questionnaire in which participants could also write any additional information they wanted to give on up to four items.
2. Component 2: Feedback of patients' responses (component 1) to CNS and their reporting of any action taken by services.

#### Work with staff before the feasibility trial

We met CNS staff to discuss the objectives of the feasibility trial, hear any concerns, clarify its purpose and stress its importance. We explained that we would first ask patients to complete the continuity assessment, and then, with their permission, pass on their responses to the CNS involved in their care. Nurses were asked to complete a clinical feedback form briefly detailing any action they had taken in response (see trial arms below).

### Feasibility randomised controlled trial

We obtained ethical approval for this part of the study from East London and the City Local Research Ethics Committee 1 on 30 May 2007. Our objectives were to:

1. Decide on appropriate outcome measures for a Phase III trial.
2. Explore changes in such outcomes in the trial.
3. Obtain professionals' and patients' views on the acceptability and practicality of delivering and receiving components of the intervention.
4. Demonstrate acceptable recruitment and retention rates.

### Recruitment

Patients were recruited from outpatient clinics and the chemotherapy suite of one NHS Trust in North London from 12 June to 25 October 2007. Patients were eligible for inclusion if they had breast, lung or colorectal cancer, had reached the end of first treatment, were able to give informed consent and were aged over 17 years.

#### Randomisation

An independent statistician used a blocked randomised design to achieve equal numbers in each trial arm. Researchers telephoned the trial centre to receive each participant's allocation from an administrator independent of the trial. The researchers were masked to block size, but not to patient allocation.

#### Trial arms

All patients received usual care.

Arm 1: No continuity intervention.

Arm 2: Completed the continuity assessment (the partial intervention).

Arm 3: Completed the continuity assessment plus their responses were fed back to the CNS who were expected to take action as necessary in any areas highlighted by patients for further attention (the full intervention). This could involve discussion with patients and/or discussion between members of the team. We did not indicate how or when actions should be taken; rather we left that to the CNS's expertise. Because our discussions with CNS in the preparatory phases of the study suggested that obtaining documentation on their actions would be difficult, we designed clinical feedback forms that were as brief and simple to complete as possible.

#### Baseline assessments

Patients in trial arm 1 completed no assessments at baseline. Patients in trial arms 2 and 3 completed a need assessment (the supportive care needs survey, (SCNS), which covers psychological, physical, sexuality, patient care and health system domains (Bonevski *et al*, 2000)). They also completed the same visual analogue scales to measure satisfaction that we had used in our earlier cohort study (King *et al*, 2008). No standardised scales exist to measure satisfaction with continuity of care, and thus visual analogue scales are a viable alternative and are particularly useful for measuring change over time in each individual.

#### Follow-up assessments

Patients in all three arms completed the continuity assessment (primary outcome), and the needs assessment and satisfaction rating (secondary outcomes) by post after 6 weeks. Non-responders were sent one reminder after 2 weeks.

#### Statistical analysis

We evaluated the randomisation by comparing centre and patients' demographic and clinical characteristics in each trial arm. We compared baseline data with the different components of the intervention in trial arms 2 and 3. Data from clinical feedback forms in arm 3 were analysed descriptively. Linear and logistic regression models were used to examine associations between covariates and outcomes at baseline. Responders and non-responders at follow-up were compared using logistic regression. Differences in outcome between arms 1 and 2 were examined using multivariable linear regression for continuous outcomes and logistic regression for binary outcomes. Where there were no differences, arms 1 and 2 were combined into one comparison group for further analysis. Differences between intervention and comparison arms were then examined using linear and logistic regression as above. We did not estimate sample size for this feasibility trial. Rather, we estimated the likely effect sizes of the intervention (arms 1 *vs* 2 and arms 1 *vs* 3) to guide sample size estimations for a full-scale randomised trial. We did not have sufficient power to examine the effect of covariates, such as degree of advanced cancer or location. However, we used multivariable regression to explore further the relationship between continuity, sociodemographic characteristics and SCNS needs for care at baseline and outcome in participants in trial arms 2 and 3.

## Results

### Clinic observations

Twenty-nine clinics were observed (six breast, 13 lung and 10 colorectal oncology outpatient clinics). Three main themes emerged from our clinic observations:

1. Continuity of care appeared to be considered with most patients.
2. The CNS is most likely to influence continuity.
3. Issues of continuity did not vary greatly between NHS Trusts or tumour types.

### Multidisciplinary team meeting observations

Thirty-two meetings were attended (10 breast, 13 lung and nine colorectal). Four main observations emerged:

1. Meetings vary in structure and content according to tumour type and NHS Trust.
2. Meetings may be dominated by individuals with strong personalities.
3. Meetings vary in use of technology, such as viewing electronic records and radiological and pathological findings.
4. Continuity of care is addressed occasionally.

### Staff interviews

Twelve healthcare professionals were asked for their views on version 1 of the continuity assessment. Few clinicians felt that multidisciplinary team meetings were the appropriate venue for consideration of this assessment mainly because:

1. Of insufficient time and staff resistance to the extra responsibility.
2. Several professionals would not know the patient in any personal way.
3. Multidisciplinary team meetings were considered too medical.

Clinicians also felt their work load would not accommodate the time it would take to complete the assessment with the patient.

### Patient interviews

Thirty-eight patients were interviewed about the revised, patient-centred continuity assessment. Forty-five percent of patients thought that most of the items were very important and relevant, whereas the remaining 55% regarded the items in varying degrees of less importance. The main issues patients felt were overlooked concerned service delivery and organisation, such as waiting times, difficulties with appointments and communication between services. The majority of patients (35 out of 38) thought that both the length and content of the assessment were adequate and none were opposed to any item. Four patients suggested that there should be more specific items on the emotional aspects of the patients' cancer experience. One participant suggested that there should be an item touching on the consequences of how one's ‘structure of life’ changes after diagnosis, for example, having to depend on a spouse. A few patients considered some items too complex to respond to simply with a ‘yes’ ‘no’ or ‘cannot say’. Most of the participants thought the assessment should be administered towards the beginning of treatment, although not too soon after diagnosis (before patients can cope emotionally), and not so late that potential gaps in continuity would be missed, and most of them recognised that they needed time to get used to service and come to terms with their emotional and service-oriented needs.

### Pretrial work with CNS

Clinical nurse specialists often regarded the continuity issues in the questionnaire as covered already or generating extra work, and some thought that the responses might be perceived as criticism of their role. These results suggested that we might have difficulty ensuring documentation of the clinical response component of the intervention in the feasibility trial.

### Feasibility trial

Of the 145 patients assessed for eligibility, 26 (18%) did not meet the inclusion criteria and 28 (19%) refused to participate. Main reasons for refusal were unable to give informed consent (poor English or illiteracy); not interested; too frail or unwell; too worried about their current clinic visit and visual impairment. This resulted in 93 patients (64% of those assessed) randomised into the study. There were no significant differences in patient characteristics between the three arms of the trial. The mean age overall was 59 years. Almost half (45%) of the patients had breast cancer, which is reflected in the high proportion of women in the trial (73%). Just over half (55%) of patients were White British and 86% spoke English as a first language. Over half (56%) of the patients were partnered, with 63% educated beyond the GCSE level, which is a basic school education. Of the 57 patients in trial arms 2 and 3 who completed a continuity assessment at baseline, 43 (74%) indicated at least one area of poor continuity, but of these, only 13 (30%) ticked the box indicating that they wanted to discuss issues further with their CNS. Patients in arms 2 and 3 who did not wish to discuss their continuity difficulties at baseline had significantly higher SCNS physical needs.

### Response to follow-up

**Figure 1:**
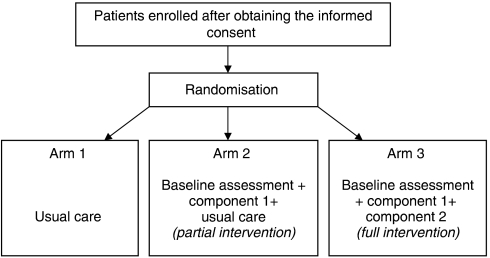
Flow diagram of the trial.

**Figure 2:**
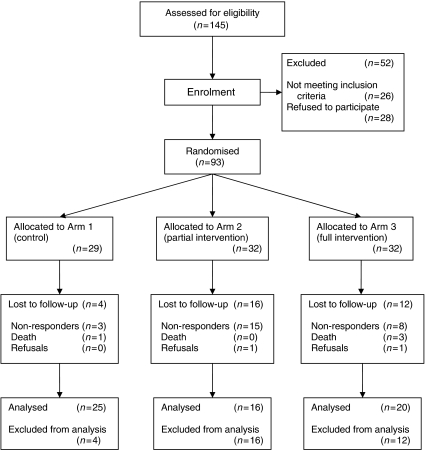
Consort diagram of the flow of the trial.

A total of 61 (66%) patients completed the outcome assessments. Attrition was highest in the 32 patients randomised to the partial intervention arm only (arm 2, Figures 1 and 2). Most (81%) of those lost to follow-up were patients who failed to respond. A small number died, and two refused to complete the follow-up questionnaires. Patients in the intervention groups were significantly more likely to drop out than those in arm 1 (arm 2: OR: 6.25, *P*=0.004, arm 3: OR: 3.75, *P*=0.042). Those with GCSE level education were significantly more likely to drop out than those with degree level education (OR: 2.98, *P*=0.042).

### Differences between trial arms at follow-up

Experienced continuity, as measured by the patient completed continuity assessment (component 1 of the intervention) was high, with a mean score of 15 (17 is the highest score possible) and varied little between the three trial arms (Table 1 ). Satisfaction, as measured by the VAS, was generally high with an average score of 82%, which differed little between trial arms. There was a clear trend towards lower scores in all categories of the SCNS (psychological, physical, health system, patient care and sexuality) from the control group that had the highest level of unmet need to the full intervention arm that had the lowest level of need (Table 1) at the follow-up.

At follow-up, patients in the intervention arms were more likely to request further discussion of issues than those in the control arm. Not all documentation of CNS activity in response to the continuity assessment was received (15 of 30 were returned), and thus for many patients, we do not know what extra action was taken or if the continuity assessment affected CNS input to their care.

### Further assessment of patients' experienced continuity, SCNS needs and satisfaction

We further examined participants in trial arms 2 and 3 who completed assessments at baseline and follow-up. These could be grouped on the basis of their responses to the continuity assessment at baseline:

1. No difficulties highlighted on the continuity assessment (14 patients).
2. Participants indicated difficulties but did not want to discuss them with staff (30 patients).
3. Participants indicated difficulties and wanted to discuss them with staff (13 patients).

Non-white patients were more likely to indicate continuity difficulties, but were less likely to want to discuss them further (Table 2). A similar association was found for language status. Patients who indicated at baseline that they wanted to discuss continuity issues further had higher SCNS physical needs at follow-up than those who had noted difficulties, but had not wanted to discuss them (Table 3). This was after adjustment for baseline SCNS needs. Patients who had not wanted to discuss their continuity difficulties at baseline had higher SCNS psychological needs and lower satisfaction at follow-up than those who had no continuity difficulties at baseline (Table 3).

## Discussion

### Main findings

We used findings from our earlier research, as well as staff and patients' views, to develop and refine our intervention to improve continuity, which was then tested in a feasibility trial. Three quarters of patients assessed at baseline (trial arms 2 and 3) indicated at least one area of poor continuity, but only one-third of these patients indicated they wanted to discuss issues further with their CNS. The full intervention (arm 3) involved patients' assessment of their own care, which was fed back for action by CNS. Although there was no measurable change in experienced continuity, there were consistent trends in needs for care in favour of the intervention arms, with those in the full intervention expressing least unmet needs for care. This mirrors our findings in earlier research where high experienced continuity was associated with lower needs for care (King *et al*, 2008). Some patients (particularly non-white people and those whose first language was not English) were more likely to indicate that they had continuity difficulties, but less likely to want them conveyed to staff. Participants who had wanted to discuss their continuity difficulties at baseline seem to have generally poorer outcomes than those who did not want to discuss them or had no continuity difficulties.

### Strengths and limitations

The main strength of this study was our ability to combine findings on continuity from our earlier work (King *et al*, 2008) with direct observation of clinics and multidisciplinary team meetings and interviews with staff and patients. The latter gave us data on the nature and content of discussions about continuity of care as well as an opportunity to ascertain how best to measure continuity of care, and implement interventions in these settings. The work also included further development of a measure of continuity and a pilot randomised controlled trial exploring feasibility of increasing experienced continuity in a clinical setting.

There were also a number of limitations. Our trial was of low statistical power as its main aim was to assess whether a randomised evaluation of a continuity intervention was possible in this setting. However, the data are an important indication of the sorts of changes we might expect if this evaluation were to be rolled out in a phase III randomised trial. The findings themselves will also be useful in future systematic reviews of trials to evaluate continuity interventions. It is important to publish the results of small trials because the data, although not significant in their own right, will contribute to the findings of a subsequent meta-analysis. Not to publish them contributes to the publication bias encountered in many systematic reviews.

Despite our finding that patients who received the partial intervention (trial arm 2) showed a trend towards poorer outcomes than those receiving the full intervention (trial arm 3), we were unable to collect consistent information on input by CNS staff. Receipt of assessments from patients may indeed have altered staff behaviour, whether or not it was recorded or fed back to the research team.

The commonest limitation expressed by staff was lack of time due to pressure of work. Complaints of lack of time when organisational change is suggested may be a consequence of staff feeling undervalued, disempowered or not heard by senior staff (Iles and Sutherland, 2001). The difficulties perceived by CNS in this trial may reflect deeper problems relating to their perceived role within the multi-disciplinary team.

It is not clear why attrition was highest in arm 2 (partial intervention). It cannot be due to repeated requests, as this would have affected arm 3 similarly. It is possible that participants in arm 2 expected that something would be done in response to completing the assessment, and when that was not the case, decided they did not want to continue in the study. This may also have been the explanation for why participants who had wanted to discuss their continuity difficulties at baseline seem to have poorer outcomes than those who did not want to discuss them or had no continuity difficulties. It may have occurred because their expectations that something would happen were not met.

Although based on our earlier research across three London cancer networks (King *et al*, 2008), this phase II work was conducted in only one network in London. Cancer services nationally work to agreed standards and targets set by the Department of Health, and are subjected to frequent peer review. However, London healthcare has been observed to be less adequate than other parts of the United Kingdom in terms of quality of care achieved (Darzi, 2007).

### Theoretical model

Our findings support the underlying theoretical framework that arose from our earlier work (King *et al*, 2008), namely that (1) patients' experience of continuity is an outcome of service delivery that can be facilitated rather than provided by professionals; (2) patients should be enabled to take control of their continuity and ensure that they feel supported and ‘in contact’ with services between appointments and (3) staff should receive feedback from patients on their experienced continuity so that they can respond in whatever clinical manner fits with their knowledge, experience and training. We now need to take that model forward with a broader evaluation.

### Recommendations for further work

Given that we showed important trends towards lower needs for care for all patients who received the full intervention, targeting only those patients reporting less than adequate continuity at baseline might show even greater effects in a full trial. It could be argued that in a phase III randomised trial, the intervention might best be targeted at patients who have continuity difficulties and a desire that these difficulties are fed back to staff. This might also mean that CNS would be more responsive in giving feedback on their actions to the trialists as they would only have to review the continuity assessments of patients with difficulties rather than all patients, as was the case in trial arm 3. However, such a conclusion may be premature. As we have discussed, only one-third of patients who had indicated continuity difficulties stated that this should be fed back to their CNS, and this desire was lowest in black and minority ethnic patients and those whose first language was not English. Thus, we might need first to encourage all patients to venture such feedback, because it is in their best interests to do so, but also particularly to encourage patients in vulnerable groups to do so.

Our results also show how important it is to engage all members of staff who are likely to be affected. Although we made considerable efforts to engage staff, greater staff education and support would be essential for a definitive trial. Staff at all levels should understand the background and importance of the research, as well as the likely benefits for patients. Engaging an influential clinician who might champion the innovation within each participating service would be a considerable help. A recent systematic review concluded that opinion leader interventions reduce non-compliance with desired clinical practice. However, identifying appropriate leaders can be labour intensive and unreliable (Doumit *et al*, 2007). Nevertheless, given that continuity of care is a central plank of the Cancer Reform Strategy (DOH, 2007), promotion of internal leadership should be possible. Robust research into improving continuity is essential to inform future policy. There is evidence that in long term, life-threatening illnesses, such as cancer, preservation of trust and hope within the professional-patient relationship, is crucial in determining whether or not patients make best use of effective treatments (Barnes *et al*, 2007). Our results show that attention to experienced continuity may lead to better patient outcomes. They also show that the key barriers to efforts to improve continuity are organisational and patient centred. By the latter, we mean the hesitation on the part of some patients to report their continuity difficulties to the service. We cannot be sure whether this is because they do not regard reporting them as helpful or whether they fear criticising the service. Nevertheless, we believe that a patient-led continuity intervention of this sort is workable. We also consider that the key staff members, such as CNS, are well placed to do this work and they felt so too. However, they were stressed with workloads, and we believe that if this was seen as a priority by the service, it might take off. Receiving back 50% of CNS' feedback forms indicates a degree of success, given the lack of any clinical champion for continuity in the services.

## Figures and Tables

**Box 1:** Statements on experienced continuity of care

**Item no.**	**Items**
1	I have received enough time and attention from the cancer services.
2	I feel I am seeing the cancer services often enough.
3	I am getting consistent information about my illness from health care staff.
4	I frequently have to chase up cancer services to get things done.
5	I have been well informed about what my treatment will involve over the next few months.
6	I feel out of touch with the cancer services between appointments.
7	I feel I am supported by the people closest to me.
8	I feel the people closest to me are able to cope with my illness.
9	I am worried about the emotional state of the people closest to me.
10	I feel I depend too much on the people closest to me.
11	I have received some misleading information from the cancer services.
12	I am satisfied that I have received a full medical examination with regard to cancer.
13	I am worried that some things may have been overlooked.
14	I know I have a specific person at the hospital whom I can contact when I need to.
15	I know how to contact this person.
16	The last time when I was in clinic, I think the clinical staff had all my notes.
17	I feel I am able to manage between appointments.

**Table 1:** Outcome measures at 6-week follow-up

	**Arm 1**	**Arm 2**	**Arm 3**	**Total**
*SCNS*^a^ *mean (s.d.)*				
Psychological	45 (34)	37 (19)	32 (28)	39 (29)
Physical	41 (32)	33 (27)	29 (25)	35 (29)
Health system	34 (24)	26 (16)	22 (19)	28 (21)
Patient care	28 (22)	25 (20)	20 (17)	24 (20)
Sexuality	23 (26)	19 (21)	18 (29)	20 (25)
Satisfaction^b^ mean (s.d.) score	83 (19)	83 (19)	80 (23)	82 (20)
Continuity assessment score (mean s.d.)	15 (4)	15 (3)	15 (3)	15 (3)
At least one difficulty reported on continuity assessment	11 (38%)	19 (59%)	15 (47%)	45 (48%)

SCNS=supportive care needs survey s.d.=standard deviation.

a SCNS is scored out of 100; 100 indicating high level of need.

b Satisfaction, as measured by the visual analogue score, is scored out of 100, higher scores indicating a higher level of satisfaction.

c The continuity assessment, is scored out of 17, 17 indicating good continuity

**Table 2:** Demographic characteristics in relation to continuity at baseline for trial arms 2 and 3

	**No difficulties highlighted on the continuity assessment**	**Participants indicated difficulties but did not want to discuss them with staff**	**Participants indicated difficulties and wanted to discuss them with staff**	**Statistics**
*Ethnicity*				
White-British	10 (36)	10 (36)	8 (29)	X^2^ (2df)=7.27
Other	5 (18)	20 (71)	3 (11)	*P*=0.026
				
*First language*				
English	15 (31)	22 (46)	11 (23)	Fisher's exact test
Other	0	8 (89)	1 (11)	*P*=0.036

Number (%).

**Table 3:** Relationship between response to continuity assessment at baseline (trial arms 2 and 3) and outcomes

	**Regression coefficient**	**95% confidence interval**		***P*-value**
*Physical needs SCNS*				
3 *vs* 2^a^	−15	−28	−2	0.024
				
*Psychological needs SCNS*				
2 *vs* 1	29	10	49	0.004
				
*Satisfaction*				
2 *vs* 1	−20	−34	−5	0.011

SCNS=supportive care needs survey.

a 1. No difficulties highlighted on the continuity assessment (14 patients). 2. Participants indicated difficulties but did not want to discuss them with staff (30 patients).

3. Participants indicated difficulties and wanted to discuss them with staff (13 patients).
